# Case series of intravenous thrombolysis for acute ischemic stroke in confirmed COVID-19 patients: single-centre experience

**DOI:** 10.1186/s41983-021-00434-w

**Published:** 2022-01-10

**Authors:** Ahmad Sulaiman Alwahdy, Ika Yulieta Margaretha, Kenyo Sembodro Pramesti, Nailaufar Hamro, Viska Yuzella, Fitriani Nasution, Arfan Mappalilu

**Affiliations:** 1 Department of Neurology, Fatmawati Central General Hospital, Jl. RS. Fatmawati Raya No.4, RT.4/RW.9, Cilandak Barat, Kec. Cilandak, Kota Jakarta Selatan, Daerah Khusus Ibukota Jakarta, 12430 Indonesia; 2 University of UIN Syarif Hidayatullah, Tangerang, Indonesia

**Keywords:** Ischemic stroke, Intravenous thrombolysis, Rt-PA, COVID-19, Case series

## Abstract

**Background:**

Coronavirus disease 2019 (COVID-19) not only caused a large surge of respiratory infections, it also had a potential association with and increases the risk of stroke. The pandemic has certainly provided new challenges and opportunities in the management of acute ischemic stroke (AIS); however, data regarding outcomes of intravenous tissue plasminogen activator (IV TPA) administration in stroke patients with COVID-19 remains limited.

**Case presentation:**

Three AIS patients with confirmed COVID-19 treated using IV tPA. One case had excellent outcome, while the other cases showed unfavorable results. The risk–benefit ratio of IV TPA in COVID-19 remains unclear.

**Conclusion:**

In this article, we discuss the possible explanation behind these different outcomes. Although IV tPA could not cure COVID-19, we suggest that its administration should not be delayed in AIS patients with COVID-19.

## Background

Coronavirus disease 2019 (COVID-19) was first reported in Wuhan, China, back in December 2019. The disease, an infectious disease caused by the severe acute respiratory syndrome coronavirus-2 (SARS CoV-2), was then declared as a pandemic by the World Health Organization (WHO) on 11 March 2020 [1]. COVID-19 patients can present with acute cerebrovascular disease. A multinational observational study received data from 26,175 hospitalized SARS-CoV-2 patients from 99 tertiary centers in 65 regions in 11 countries. From a total of 17,799 SARS-CoV-2 infection, 156 patients reportedly had stroke. Among the 156 stroke patients, 123 (79%) presented with Acute Ischemic Stroke (AIS), 27 (17%) with intracerebral/subarachnoid hemorrhage, and 6 (4%) with cerebral venous or sinus thrombosis [2, 3].

COVID-19 is associated with hypercoagulability, dysregulated immune response leading to cytokine-release syndrome, and with damage to endothelial cells leading to increased inflammation and thrombosis [4]. It has also been associated with increased rates of arterial and venous thromboses, both systemic and in the pulmonary vasculature [5]. Another aspect to consider is hepatic dysfunction which often manifests as elevation of transaminases levels in patients with COVID-19 [6]. Currently, there is no specific guidelines regarding IV thrombolysis in AIS patients with confirmed COVID-19.

Acute ischemic stroke treatment using intravenous rtPA is recommended for selected patients who can be treated within 3 h and for highly selected patients who can be treated within 3 and 4.5 h of symptom onset. Intravenous rtPA undergoes hepatic clearance which may be reduced in hepatic dysfunction, leading to a potential increase of serum levels and an increased risk of intracranial hemorrhage (ICH). Hemorrhagic Transformation (HT) is the most serious complication of intravenous thrombolysis in AIS. The incidence rate of symptomatic intracranial hemorrhage is 2.2–8% worldwide in non-COVID patients [7, 8]. Therefore, advanced hepatic dysfunction may be associated with coagulopathy with elevation in prothrombin time (PT), international normalized ratio (INR), and thrombocytopenia; thus, a detailed assessment of coagulation profiles to determine the risk:benefit ratio is preferable prior to intravenous rtPA administration, especially in COVID patients [3, 9].

In a study by Deppa, et al., improvement of the PF (PaO_2_/FiO_2_) ratio following alteplase may reflect both improvement not only in AIS but also in alveolar perfusion and ventilation. In the absence of significant bleeding with preserved fibrinogen levels 24 h post-thrombolysis, a significantly improved PF ratio in this report suggests that fibrinolysis may be beneficial in a carefully selected group of patients with close monitoring [8, 9].

## Case presentation

### Case 1

**Fig. 1:**
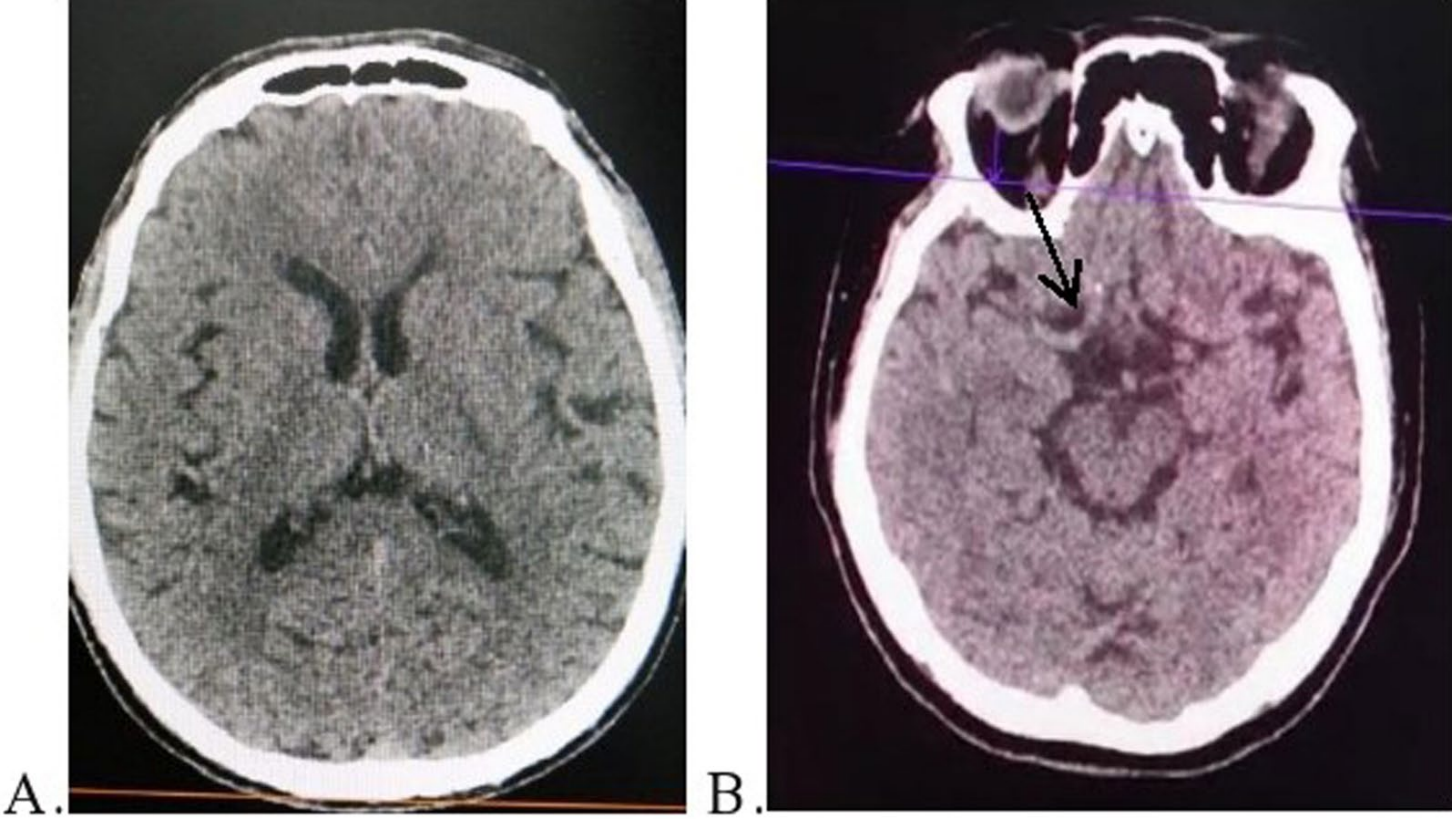
Imaging of ischemic stroke patients with COVID-19. **A** CT brain from case 1, showing hypodensities in left ganglia basal, insula and internal capsule. **B** Case 2, hypodensities were not found, MCA dense sign were prominent (black arrow). *MCA* middle cerebral artery

A 62-year-old man with a history of hypertension and prediabetes was admitted 2.5 h after the onset of right hemiparesis and aphasia. After administering the National Institutes of Health Stroke Scale (NIHSS), the patient was found to have a score of 12. Vital signs on presentation were as follows: heart rate 90 beats/min; blood pressure 165/85 mmHg; and respiratory rate of 24 breaths/min. Saturation of blood oxygen (SpO_2_) was 90% on room air in the emergency department and improved to 98% by nasal cannula supplementation of 5 L/min. We performed brain Computed Tomography (CT) scan (Revolution EVO, GE Healthcare 128, United States) which revealed subtle hypodensity in left basal ganglia, left insula, and left internal capsule with an ASPECT score of 7 (Fig. 1a). He was diagnosed with acute ischemic stroke and IV tPA was initiated at a dose of 0.9 mg/kg body weight 4 h post-onset with 10% bolus of the total dose given as initial loading, followed by administration of the remaining dose as an infusion over 1 h. During the infusion, patient developed gum bleeding, and the infusion was stopped (80% of the total IV tPA dose had been administered). Chest X-ray showed suspected viral infection and nasopharyngeal swab was positive for SARS-CoV-2 RNA. Laboratory examination was also performed, revealing changes in coagulation markers and slight increase of c-reactive protein (CRP) (Table 1). The patient did not have fever or shortness of breath and only had a mild cough. Treatment for COVID-19 was initiated using favipiravir according to our hospital protocol, along with antibiotic and subcutaneous enoxaparin 40 mg once daily. The following day, deficits progressively improved with an NIHSS of 4 and the patient was eventually discharged.

**Table 1:** Clinical and demographical features of three patients

	Patient 1	Patient 2	Patient 3
Age	62	59	58
Sex	Male	Female	Female
Patient history			
Pre-stroke mRS (0–5)	0	0	0
Comorbidities	Hypertension, prediabetic	Hypertension, Diabetic, Obesity	Hypertension, Obesity
Antiplatelet/anticoagulant	No	No	No
COVID-19 characteristics			
Nasopharyngeal swab	Positive	Positive	Positive
Chest X-ray	Viral pneumonia characteristics (bilateral)	Viral pneumonia characteristics (bilateral)	Viral pneumonia characteristics (bilateral)
Fever	No	Yes	No
Cough	Yes	No	No
Dyspnea	No	Yes	Yes
Myalgia	No	No	No
Gastrointestinal symptoms	No	No	No
Pharmacological treatment	Favipiravir, antibiotic therapy; Levofloxasin, Azithromycin and enoxoparin 40 mg once daily	Favipiravir, antibiotic therapy; Levofloxasin, Ceftriaxone and enoxoparin 40 mg once daily	Favipiravir, antibiotic therapy; Levofloxasin, Ceftriaxone. Enoxoparin (-)
IV tPA	Yes (80% of the total IV tPA dose)	Yes	Yes (80% of the total IV tPA dose)
Initial SpO_2_ (room air)	90%	80%	93%
Oxygen therapy	Nasal Cannule	NRB	NRB
Laboratory findings			
Haemaglobin, g/dl	15.4	11	13.8
Leucocyte × 10^9^/L	7.7	10.2↑	11.8↑
Platelets × 10^9^/L	538↑	516↑	314
Neutrophils, %	71↑	89↑	78↑
Lymphocytes, %	12↓	6↓	15↓
aPTT, s	26	27	28.1
PT, s	13.4	13.8	13.4
INR	0.94	0.97	0.92
Fibrinogen, mg/dl	473↑	655↑	423↑
D-dimer, ng/ml	1710↑	> 20,000↑	910↑
AST, U/L	31	13	15
ALT, U/L	58↑	18	9
Creatinine, mg/dl	–	0,4	0.8
Glucose, mg/dl	168↑	410↑	140
LDH, U/L	–	813↑	–
CRP, mg/dl	0.7	8.3↑	2.9↑
Stroke features			
Clinical symptoms	Right hemiparesis, aphasia	Left hemiparesis, dysarthria	Right hemiparesis,dysarthria
NIHSS at baseline	12	10	10
NIHSS after 24 h	4	6	Not available
Haemorrhagic transformation	No	No	Yes (Intraventricular haemorrhagic)
Complication	None	Respiratory insufficiency (ARDS)	Respiratory insufficiency (ARDS)
Outcome	Clinical recovery	Death	Death

*ALT* alanine aminotransferase, *aPTT* activated partial thromboplastin time, *ARDS* acute respiratory distress syndrome, *AST* aspartate aminotransferase, *COVID-19*coronavirus disease 2019, *CRP* C-reactive protein, *IV tPA* intravenous tissue plasminogen activator, *INR* international normalized ratio, *LDH* lactate dehydrogenase, *mRS* modified ranking scale, *NIHSS* National Institutes of Health Stroke Scale, *spo*_*2*_oxygen saturation, *PT* prothrombin time

### Case 2

A 59-year-old diabetic woman with a history of hypertension was admitted for left hemiparesis and moderate dysarthria 2 h prior to admission. Her NIHSS score on presentation was 10. Vital signs on presentation were as follows: temperature 37.5 °C; heart rate 98 beats/min; blood pressure 140/80 mmHg; and respiratory rate of 28 breaths/min. SpO_2_ was 80% on room air in the emergency department and increased to 88% using nasal cannula O_2_ supplementation, with 100% fraction of inspired oxygen (FiO_2_) on a non-rebreathing mask (NRB). We performed brain CT scan (Revolution EVO, GE Healthcare 128, United States), which showed no marked hypodensities. However, the right middle cerebral artery (MCA) was prominently suspected for large vessel occlusion (LVO) (Fig. 1B). Chest X-ray revealed signs of viral infection and probable COVID-19 infection. Due to overload of patients in our emergency room and the increased number of COVID-19 patients during the pandemic, the result of nasopharyngeal swab in this patient was delayed. No endovascular treatment was performed. Intravenous tPA was initiated at a dose 0.9 mg/kg body weight 3.5 h post-onset with 10% bolus of the total dose given as initial loading, followed by administration of the remaining dose as an infusion over 1 h. The deficits progressively improved, as indicated by NIHSS score of 6 assessed on the same day. Surprisingly, her SpO_2_ improved to 93%. Her laboratory findings were not as favorable as our first patient (Table 1) and nasopharyngeal swab result was positive for SARS-CoV-2 RNA. Favipiravir, antibiotic and subcutaneous enoxaparin 40 mg once daily were initiated. On day 3, her saturation dropped sharply to 60%. Unfortunately, due to overcapacity from COVID-19 patients surge in our hospital, intubation could not be performed. The patient’s condition continued to worsen into acute respiratory distress syndrome (ARDS). As the patient was a do-not-resuscitate patient, we performed no resuscitation, and her time of death was declared shortly after.

### Case 3

**Fig. 2:**
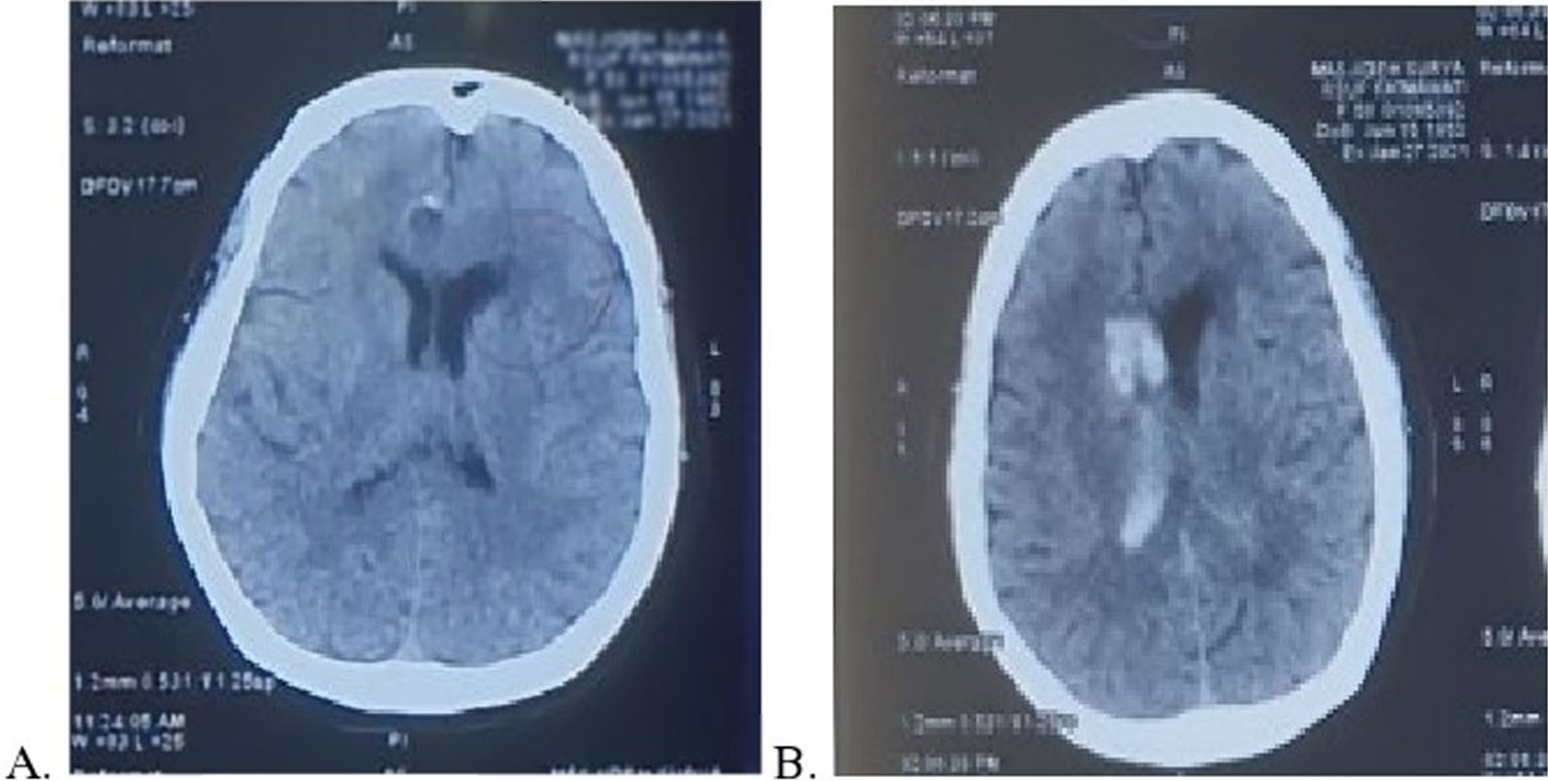
Head CT Scan **A** Before thrombolysis showed infarct in capsula eksterna sinistra cornu anterior, **B** After thrombolysis showed bleeding in lateral ventricle bilateral especially in the right and third ventricle

A 58-year-old female came to our emergency department with complaints of right-sided arm and leg weakness since 2 h prior. She also had speech difficulty and dizziness. Other symptoms such as fever, nausea, vomiting, cough, and facial droop were denied. She had a history of stroke in 2012 and uncontrolled hypertension treated with 5 mg Amlodipine. Upon physical examination, her vital signs were as follows: blood pressure 190/120 mmHg, heart rate 94 beats/min, respiratory rate 20 breaths/min, SpO_2_ 93%, and her temperature was 36.2 °C. On neurological examination, the patient had a score of 15 on the Glasgow Coma Scale (GCS). Muscle strength was 1/5 in both upper and lower right extremities. Other examinations were within normal limit. For this patient, we performed brain CT-Scan (Revolution EVO, GE Healthcare 128, United States) prior to thrombolysis (Fig. 2A). The result of polymerase chain reaction (PCR) swab test for SARS-CoV-2 was positive.

Nicardipine drip was given to reduce her blood pressure and after 10 min, the blood pressure decreased to 175/102 mmHg and IV tPA was initiated. Unfortunately, at the end of IV tPA (80% dose) there was sudden elevation of patient’s blood pressure to 200/100 mmHg and she complained of severe headache, resulting in the termination of thrombolysis. Another brain CT-Scan was immediately performed after thrombolysis (Fig. 2B) and we decided to performed an external ventricular drainage (EVD). After the surgery, the patient recovered well, was conscious and had stable blood pressure. Unfortunately, 2 days later, the patient developed ARDS and could not be saved.

## Discussion

**Fig. 3:**
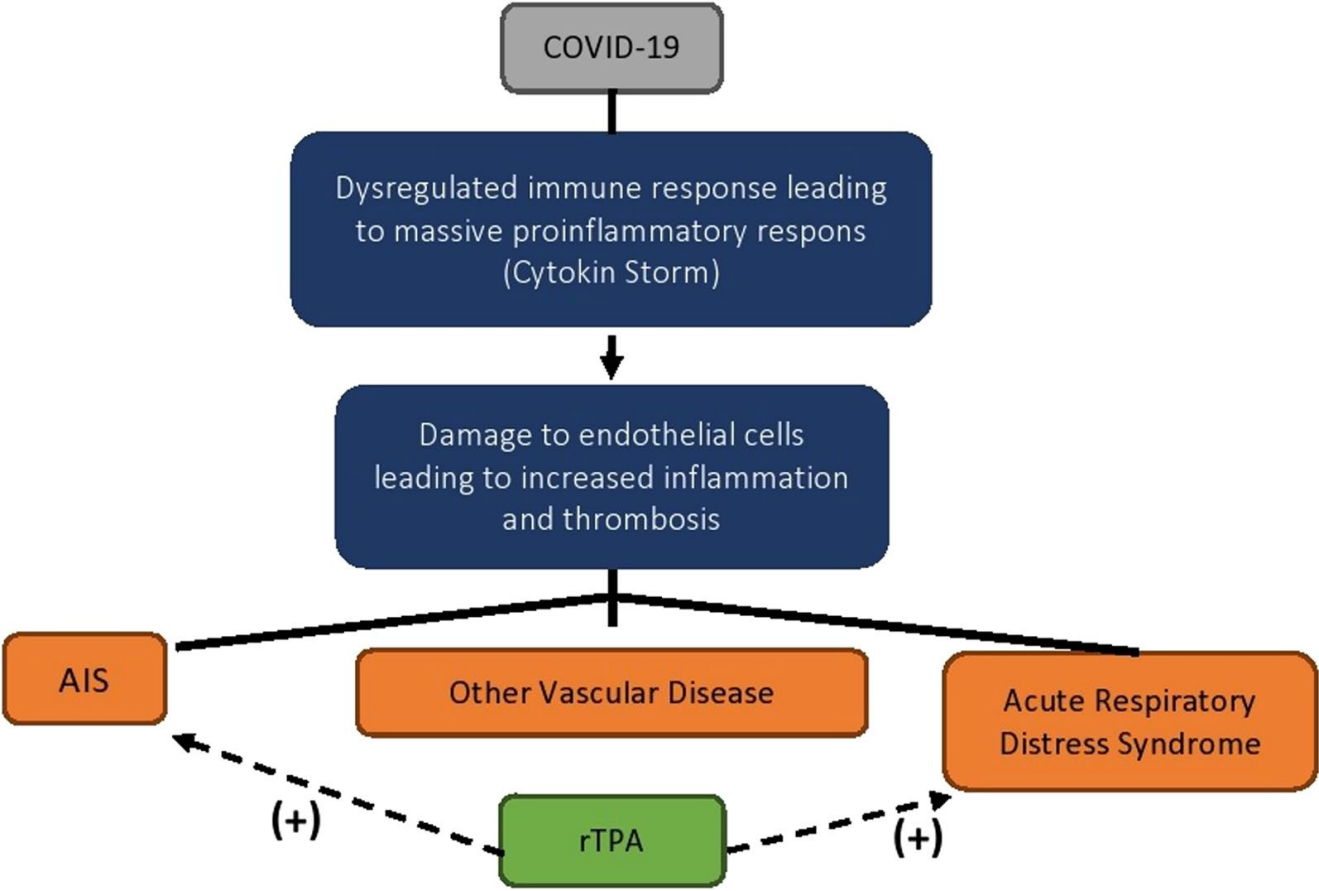
Possible mechanism of thrombosis and effect of trombolysis drugs in COVID-19

COVID-19 is a disease caused by an infection of SARS-CoV-2. Once viruses bind to the host’s receptors (attachment), they enter host cells through endocytosis or membrane fusion (penetration). Once the viral contents are released inside the host cells, viral RNA enters the nucleus for replication. Then, new viral particles are made (maturation) and released. The symptoms of patients infected with SARS-CoV-2 ranges from minimal symptoms to severe respiratory failure with multiple organ failure. Dendritic cells and macrophages serve as innate immune response against viruses until adaptive immunity is involved. T cell responses are initiated by antigen presentation via dendritic cells and macrophages [10]. In addition to respiratory symptoms, thrombosis and pulmonary embolism have been observed in severe diseases. This is in line with the finding that elevated d-dimer and fibrinogen levels were observed in severe diseases. The endothelium function includes the promotion of vasodilation, fibrinolysis, and anti-aggregation; thus, the endothelium plays a significant role in thrombotic regulation [11] (Fig. 3). All our patients had high d-dimer and fibrinogen levels (Table 1). The term 'immunothrombosis' best describes the interaction between the innate immune system and thrombosis. It focuses on the interplay between activation of intravascular TF, innate immune cells, platelets, endothelial cells, and releases neutrophil extracellular traps (NETs) which can activate the coagulation cascade through the contact pathway[4].

Impaired coagulation in COVID-19 has been identified as a significant indicator of poor prognosis, and recent reports attempted to understand COVID-19-related coagulopathy and raised awareness of acute pulmonary embolism (PE) events [12]. No specific guidelines exist for the management of massive PE in COVID-19. Thrombolysis could be an effective and safe therapy for massive PE in mechanically ventilated COVID-19 patients [13]. A reported case of one COVID-19 patient with pneumonia and acute PE, infused a total of 30 mg of rtPA over 15 h, and the patient's clinical condition improved rapidly [12].

Intraventricular hemorrhage (IVH) occurs when blood from a cerebral hemorrhage expands into the brain ventricular system. The exact etiopathogenesis of IVH remains unclear. The common associations include hypertension, arteriovenous malformations (AVMs), aneurysms, moyamoya disease (MMD), coagulopathy, and arteriovenous fistula. Chronic hypertension is believed to induce hemorrhage in choroidal arteries that probably results in Primary IVH [14]. We believe that our patient’s IVH did not arise from the thrombolysis. In a retrospective clinical study involving 98 adults, reported a 66.2% incidence for IVH, with idiopathic primary IVH and hypertension considered to be the primary risk factor in 62.24% of patients [15]. Our patient had a history of uncontrolled hypertension for more than 5 years, and we, therefore, suggest that IVH in our patient occurred due to uncontrolled hypertension.

Patients with AIS and COVID-19 could still be given intravenous thrombolysis. Current international guidelines recommend intravenous thrombolysis for selected patients who can be treated within 3 h and for highly selected patients who can be treated within 3–4.5 h of symptom onset. This recommendation comes with the caveat that patients with COVID-19 have been found to have a high prevalence of elevated concentration of inflammatory and hypercoagulability markers, which have been associated with increased risk of death or disability and post-thrombolytic intracranial hemorrhage. However, in our patient, it does not seem to be related to IV thrombolysis. Furthermore, we found that rtPA could be beneficial for ARDS patients although it is temporary and depends on the progressiveness of COVID-19 [12]. Based on our experience, IV thrombolysis in AIS patients with confirmed COVID-19 should not be delayed. Evaluation of both liver function and coagulopathy markers may aid in the assessment of benefit/risk profile, and, therefore, should be performed prior to rtPA administration[1].

## Conclusions

In our experience, although IV tPA could not cure COVID-19, its administration should not be delayed in AIS patients with COVID-19. Thrombolysis could be beneficial not only for thrombolysis in the brain vessels but also in other vessels, such as pulmonary/alveolar vessels. We believed IVH in our patient did not occur due to the thrombolysis itself, but rather from an uncontrolled hypertension. Larger scale studies with more patients are needed to better elucidate the benefit of thrombolysis in COVID-19 patients with AIS.

## Data Availability

The data that support the findings of this study are available on request from the corresponding author. The data is not publicly available due to privacy or ethical restrictions.

## Abbreviations

AIS: Acute ischemic stroke
ARDS: Acute respiratory distress syndrome
ASPECT: Alberta stroke programme early CT
AVM: Arteriovenous malformations
COVID-19: Coronavirus disease 2019
CRP: C-reactive protein
CT scan: Computed tomography scan
EVD: External ventricular drainage
FiO_2_: Fraction of inspired oxygen
GCS: Glasgow Coma Scale
HT: Hemorrhagic transformation
ICH: Intracranial hemorrhage
INR: International normalized ratio
IVH: Intraventricular hemorrhage
IV tPA: Intravenous tissue plasminogen activator
LVO: Large vessel occlusion
MCA: Middle cerebral artery
MMD: Moya moya disease
NETs: Neutrophil extracellular traps
NIHSS: National Institutes of Health Stroke Scale
NRB: Non-rebreathing
PCR: Polymerase chain reaction
PE: Pulmonary embolism
PF ratio: PaO_2_/FiO_2_ ratio
PT: Prothrombin time
RNA: Ribonucleic acid
SARSCoV-2: Severe acute respiratory syndrome corona virus-2
WHO: World Health Organization
